# The molecular characterisation of *Escherichia coli* K1 isolated from neonatal nasogastric feeding tubes

**DOI:** 10.1186/s12879-015-1210-7

**Published:** 2015-10-26

**Authors:** Aldukali Alkeskas, Pauline Ogrodzki, Mohamed Saad, Naqash Masood, Nasreddin R. Rhoma, Karen Moore, Audrey Farbos, Konrad Paszkiewicz, Stephen Forsythe

**Affiliations:** School of Science and Technology, Nottingham Trent University, Clifton Lane, Nottingham, NG11 8NS UK; Wellcome Trust Biomedical Informatics Hub, Biosciences, Stocker Road, University of Exeter, Exeter, EX4 4QD UK

## Abstract

**Background:**

The most common cause of Gram-negative bacterial neonatal meningitis is *E. coli* K1. It has a mortality rate of 10–15 %, and neurological sequelae in 30–50 % of cases. Infections can be attributable to nosocomial sources, however the pre-colonisation of enteral feeding tubes has not been considered as a specific risk factor.

**Methods:**

Thirty *E. coli* strains, which had been isolated in an earlier study, from the residual lumen liquid and biofilms of neonatal nasogastric feeding tubes were genotyped using pulsed-field gel electrophoresis, and 7-loci multilocus sequence typing. Potential pathogenicity and biofilm associated traits were determined using specific PCR probes, genome analysis, and *in vitro* tissue culture assays.

**Results:**

The *E. coli* strains clustered into five pulsotypes, which were genotyped as sequence types (ST) 95, 73, 127, 394 and 2076 (Achman scheme). The extra-intestinal pathogenic *E. coli* (ExPEC) phylogenetic group B2 ST95 serotype O1:K1:NM strains had been isolated over a 2 week period from 11 neonates who were on different feeding regimes. The *E. coli* K1 ST95 strains encoded for various virulence traits associated with neonatal meningitis and extracellular matrix formation. These strains attached and invaded intestinal, and both human and rat brain cell lines, and persisted for 48 h in U937 macrophages. *E. coli* STs 73, 394 and 2076 also persisted in macrophages and invaded Caco-2 and human brain cells, but only ST394 invaded rat brain cells. *E. coli* ST127 was notable as it did not invade any cell lines.

**Conclusions:**

Routes by which *E. coli* K1 can be disseminated within a neonatal intensive care unit are uncertain, however the colonisation of neonatal enteral feeding tubes may be one reservoir source which could constitute a serious health risk to neonates following ingestion.

**Electronic supplementary material:**

The online version of this article (doi:10.1186/s12879-015-1210-7) contains supplementary material, which is available to authorized users.

## Background

Liu *et al*. [1] reported that worldwide the estimated mortality in children younger than 5 years in 2010 was 7,600,000. Neonates, in particular those with very low birth-weight, are of particular concern due to their weak immune system [2]. The risk of infection in neonates increases with low birth weight and additionally prolonged hospitalization [3, 4]. Mortality among neonates is attributed to infectious causes, preterm birth complications, intrapartum-related complications, sepsis and meningitis.

The most common cause of Gram-negative bacterial neonatal meningitis is *E. coli* K1. It has a mortality rate of 10–15 %, and neurological sequelae in 30–50 % of cases [5–8]. Other bacteria responsible for neonatal and infant morbidity and mortality include Group B streptococci (GBS), *Enterobacter* spp., *Citrobacter koseri*, *Neisseria meningitidis*, *Serratia* spp., and *Cronobacter* spp. [9, 10].

*E. coli* serotypes associated with neonatal meningitis are primarily O18:K1:H7, O1:K1, O7:K1, O83:K1 and the more recently reported O45:K1:H7 [11–13]. These are in the *E. coli* extraintestinal pathogenic (ExPEC) subgroup B2, and are sequence type (ST) 95 (Achtman scheme) [14]. Bacterial invasion across the blood–brain barrier is multifactorial, requiring several genes for binding, invasion and intracellular survival. Proposed virulence genetic determinants include *ibeA*, *sfaS*, *cnf1*, *gimA*, and *ompA* [15, 16]. However these are not always supported in experimental assays and are not demonstrable in all *E. coli* strains isolated from cerebral spinal fluid [12, 13, 17].

Although there have been some concerns regarding bacterial biofilm formation inside neonatal nasogastric feeding tubes by opportunistic pathogens, few systematic studies have been undertaken [18–20]. Mehall et al. [18] reported both feeding intolerance and a link to necrotizing enterocolitis in neonates following the bacterial colonised of their feeding tubes. Previous studies by Hurrell et al. [19] have revealed that *in situ* the inside of such tubes can be colonised by a variety of fungi and various opportunistic bacterial pathogens producing a biofilm of mixed microbial composition; *Candida* spp., *E. coli, Enterobacter hormaechei, Klebsiella pneumoniae, Cronobacter sakazakii, Yersinia enterocolitica,* and *Pseudomonas fluorescens*. The aim of this study was to investigate the diversity of the *E. coli* strains previously isolated by Hurrell et al. [19] from the residual liquid in the lumen and biofilm from 30 neonatal nasogastric feeding tubes, which had been collected from among 129 neonates on two intensive care units.

## Methods

### Bacterial strains used

Thirty isolates of *E. coli* were included in this study; Table 1. All isolates had previously been isolated by Hurrell et al. [19] from the residual lumen liquid and biofilms of nasogastric enteral feeding tubes on neonatal intensive care units at Hospital 1 (*n* = 3) and 2 (*n* = 27).

**Table 1:** Source of *E. coli* strains used in this study; adapted from Hurrel et al. [19]

MLST sequence type	Strain number	Location recovered from NGT	Hospital	Date of isolation	Neonate	Feeding source	Duration (h)	Oral antibiotics given	Gastric pH	Age (wk)	Frequency of feeding
ST2076	1047	Biofilm	1	22/10/2007	116	RTF	12–18	No	4.5	2–3	Every 2 h
	1050	Lumen			116						
	1051	Lumen			116						
ST73	1009	Biofilm	2	05/06/2007	101	BMF	>48	No	4.0	>4	Every 2 h
	1010	Biofilm		12/06/2007	102	BMF	12–18	No	NG	>4	Every 2 h
	1015	Biofilm		12/06/2007	104	BM	24–48	No	3.5	3–4	Every 2 h
	1016	Lumen			104						
ST95	904	Biofilm	2	24/04/2007	55	BM,IF	24–48	Yes	4.5	3–4	Every 3 h
	905	Lumen			55						
	926	Biofilm		01/05/2007	61	BMF, RTF	6–12	No	NG	>4	Cont.
	927	Lumen			61						
	929	Biofilm		01/05/2007	62	PIF	>48	No	5	>4	Cont.
	933	Lumen			62						
	934	Biofilm		01/05/2007	63	RTF	18–24	No	3.5	3–4	Every 3 h
	910	Biofilm		03/05/2007	64	BMF, RTF	>48	No	4	1–2	Every 2 h
	912	Lumen			64						
	913	Biofilm		03/05/2007	65	RTF	24–28	No	5.5	>4	Cont.
	917	Lumen			65						
	923	Biofilm		08/05/2007	67	BMF	12–18	No	2.5	1–2	Every 2 h
	924	Lumen			67						
	937	Lumen		08/05/2007	68	BMF, RTF	18–24	Yes	NG	3–4	Every 2 h
	939	Biofilm		08/05/2007	69	BMF	<6	No	4.5	1–2	Every 2 h
	943	Biofilm		08/05/2007	70	RTF, Th	24–48	No	4.0	>4	Every 2 h
	944	Lumen			70						
	947	Lumen		08/05/2007	71	PIF, Th	<6 h	No	3.5	>4	Every 4 h
	949	Biofilm			71						
ST127	780	Biofilm	2	16/01/2007	4	BM	18–24	No	3.5	>4	Every 2 h
	796	Biofilm		30/01/2007	17	BM, PIF	6–12	No	3–3.5	>4	Every 2 h
	786	Biofilm		17/02/2007	37	BMF, PIF, Th	18–24	No	4	>4	Every 3 h
ST394	1008	Lumen	2	06/06/2007	99	BMF, RTF, Th	>48	No	4.5	2–3	Every 3 h

*Duration* Period of time NG tube in place, *BM* Breast milk, *NG* Not given, *BMF* breast milk fortified, *Cont.* continuous feed, *PIF* reconstituted powdered infant formula, *NGT* nasogastric tube, *IF* infant formula, no further description given, *RTF* ready to feed formula, *Th* thickener added to feed

### Pulsed-field gel electrophoresis

Pulsotypes were determined using pulsed-field gel electrophoresis (PFGE) with XbaI and SpeI restriction enzymes as described by PulseNet [21]. *Salmonella enterica* serovar Typhimurium H9812 was used as the reference strain. Dendrogram construction and band assignment was achieved using BioNumerics software version 3.5. Dice coefficient, unweighted pair group method with arithmetic mean (UPGMA) were used for cluster analysis. Less than 95 % of band similarity value was used to consider the isolates to be non-clonal [22]. The tolerance and optimization of the bands was 1.5 %.

### Multilocus sequence typing (MLST)

Sequence type (ST) of the *E. coli* isolates used the 7-loci MLST Achtman scheme (http://mlst.warwick.ac.uk/mlst/mlst/dbs/Ecoli). Seven housekeeping genes were amplified by PCR using the primers for *adk* (adenylate kinase), *fumC* (fumarate hydratase), *gyrB* (DNA gyrase), *icd* (isocitrate/isopropylmalate dehydrogenase), *mdh* (malate dehydrogenase), *purA* (adenylosuccinate dehydrogenase), and *recA* (ATP/GTP binding motif). The sequences were aligned using CLC Sequence Viewer 6.6 (http://www.clcbio.com). The trimmed allele sequences were compared against the *E. coli* MLST database (http://mlst.warwick.ac.uk/mlst/mlst/dbs/Ecoli) and the sequence types were subsequently determined.

### Serotyping

The O-antigen serotype was determined using comparative genomic analysis, and confirmed by laboratory analysis (Statens Serum Institut) [23].

### Motility determination

Motility was determined by measuring the zones of growth in semi-solid agar [24]. A single colony from each strain was used to inoculate 3 ml of TSB which was then incubated at 37 °C with shaking incubator at 200 rpm. The culture was diluted to 10^4^ CFU/ml and 3 μl of the suspension used to stab inoculate TSB supplemented with 0.4 % agar. The inoculated plates were incubated overnight at 37 °C. Strains were analysed twice, each time in triplicate.

### Haemolysis reaction

Haemolysis was examined by streaking on TSA-blood agar plates containing 5 % sheep blood (Oxoid Thermo Fischer Scientific, UK), and then incubating for 24 h for at 37 °C. The resultant colony morphology was recorded after 24 h to determine the formation of either α- or β-haemolysis.

### Antibiotic resistance determination

Antibiograms were determined using the disc diffusion method. Antibiotic discs were obtained from MAST Group (UK). For each antibiotic the diameter of the zone was measured and then compared with standard measurements to determine if the strains were resistant or sensitive to the antibiotic [25]. The control strains were *E. coli* NCTC 13351, *E. coli* NCTC 13352, *E. coli* NCTC 13353 and *E. coli* NCTC 10418. The presence of the β-lactamase resistance genes *SHV*, *TEM*, *CTX-M*, and *OXA* were screened for by a multiplex PCR assay [26]. Strains with known β-lactamase types were included as reference strains. These were *E. coli* NCTC 13351 (*TEM*-3), *E. coli* NCTC 13353 (*CTX-M-15, TEM, OXA*), *K. pneumoniae* NCTC 13368 SHV-18. Genomes were analysed using the Comprehensive Antibiotic Database (CARD; http://arpcard.mcmaster.ca) for genes encoding antibiotic resistance [27].

### PCR detection of virulence factor genes

The presence of 30 virulence factor genes was determined using 5 multiplex PCR–based assays [28]. The gene classes included adhesins (*papAH, papC, papEF, papG, sfa/focDE, sfaS, focG, afa/draBC, bmaE, gafD, nfaE* and *fimH*), toxins (*hlyA*, *cnf1*, *cdtB)*, siderophores (*fyuA*, *iutA*), polysaccharide coatings (*kpsMT* II *kpsMT* III*, kpsMT* K1*, kpsMT* K5), invasins (*ibeA)* and others (*rfc, cvaC, traT, malX*).

### Attachment and invasion assay

Attachment and invasion assays to examine the capability of selected bacterial strains to attach and invade mammalian cells (Caco-2, rBCEC4 and HBMEC) were as previously described [29]. Bacterial strains were investigated for their uptake and persistence in macrophages (U937) obtained from the American Type Culture Collection [30].

### Adherence pattern determination

The Giemsa stain was used to determine the adherence pattern of the *E. coli* strains. Caco-2 and Hep-2 cell monolayers were grown on tissue culture coverslips in six-well tissue culture plates [31]. The slides were seeded with 2 × 10^4^ cells and then incubated at 37 °C with 5 % CO_2_ for 48 h. After the incubation period, the monolayers were infected with 10^8^ per well of overnight bacterial culture and further incubated at 37 °C under 5 % CO_2_ for 2 h. The coverslips were washed three times with sterile PBS, fixed with absolute methanol for 5 min and allowed to air dry. The cells were stained with 5 % of Giemsa stain (Life Technologies™, UK) for 15 min, washed with sterile PBS and allowed to air dry. The slide was examined using light microscopy.

### Genomic analysis

Bacterial DNA was extracted from 1-day old cultures of selected strains using GenElute™ bacterial genome kit (Sigma Aldrich®, USA). The genome sequences were generated on an Illumina MiSeq using v3 chemistry and 300 bp paired end reads using dual indexed Nextera XT libraries. The *de novo* assembly was performed using SPAdes assembly program and Quast [32]. Genome annotation used the SEED-based automated annotation system provided by the RAST server (http://rast.nmpdr.org) and prokaryotic genome annotation system PROKKA [33].

The genome sequences obtained were compared to published chromosomal, plasmid and O-antigen sequences for *E. coli* APEC O1 (Accession number CP000468), *E. coli* CE10 O7:K1 (Accession number GCA_000227625), *E. coli* S88 O45:K1:H7 (Accession numbers: chromosome CU928161, plasmid CU928146), O-antigens O1 and O2 (Accession numbers GU299791 and GU299792, respectively), and plasmids *E. coli* O1 pAPEC-O1-CelBM (Accession number DQ381420) and *E. coli* O18:K1 pRS218 (Accession number CP007150) [1, 13, 17, 34–37]. The genomes were also searched for various biofilm formation associated traits. Whole genome alignment used Parsnp from the Harvest Tools software v1.1.2 with a reference genome selected at random. Tree visualisation used FigTree v1.4.2, to construct a midpoint rooted tree.

### Nucleotide sequence accession numbers

The Whole Genome Shotgun projects have been deposited at DDBJ/EMBL/GenBank under accession JQFB00000000, JQFC00000000, JQFD00000000, JQFQ00000000, JQFR00000000, JQFE00000000, JQFF00000000, JQFS00000000, JQFG00000000, JQFH00000000, JQFI00000000 and JQFT00000000 for isolates 904, 910, 913, 923, 926, 929, 934, 937, 939, 943, 947 and 949 respectively.

## Results

### Pulsed-field analysis of *E. coli* strains

**Fig. 1:**
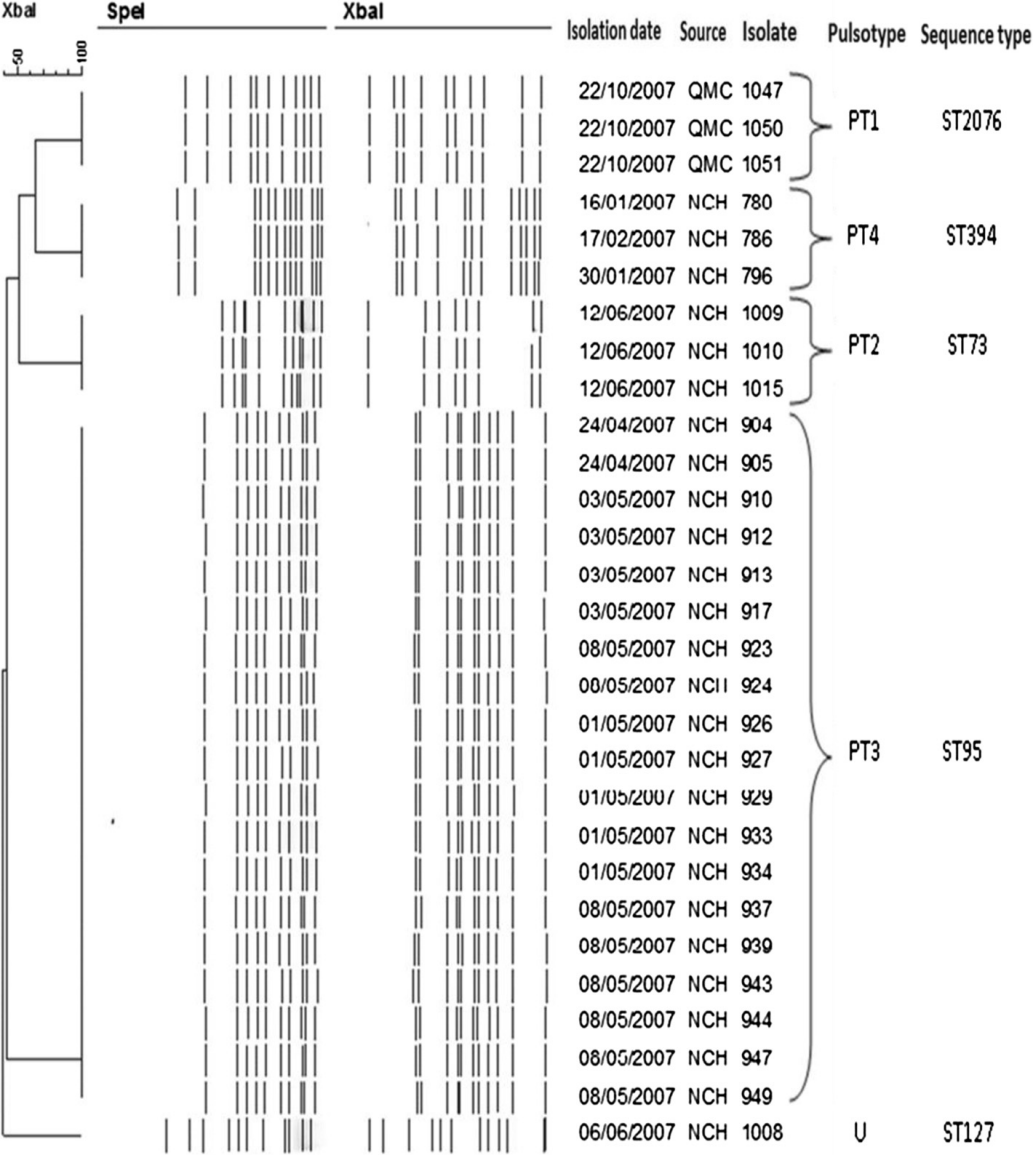
Pulsed-field gel electrophoresis of thirty *E. coli* strains isolated from neonatal nasogastric feeding tubes

As shown in Fig. 1, the PFGE analysis of thirty of *E. coli* isolates from neonatal enteral feeding tubes from hospital 1 (*n* = 3) and hospital 2 (*n* = 27) showed the strains clustered into four pulsotypes (PT1-4) and one unique (U) strain. These strains were isolated from the residual liquid in the tube and from biofilms on the inner wall of 30/129 feeding tubes (Table 1). Three strains (1047, 1050, 1051) belonging to PT1 had been isolated from the same neonate on the same day (22 October 2007). These were from both the lumen contents and biofilm within the tubes. This neonate had been fed ‘ready to feed’ formula. Four strains (1009, 1010, 1015, 1016), previously isolated from both the residual liquid and biofilm of feeding tubes, formed PT2. These had been isolated on the same day (12 June 2007) from 3 neonates fed either breast milk or fortified breast milk. Three strains (786, 780, 796) from feeding tube biofilms belonged to PT4 and were isolated over a one month period (16 January to 16 February 2007) from 2 different neonates fed breast milk, fortified breast milk, and reconstituted infant formula. There was one unique (U) strain (1008) from a neonate who had been fed both fortified breast milk and reconstituted infant formula. Of particular interest were the nineteen strains belonging to PT3. These had isolated over a two week period (24 April to 8 May 2007) from 11 different neonates; Table 1. These neonates had been fed during the sampling period breast milk, fortified breast milk, reconstituted infant formula, and ready to feed formula. Again, indistinguishable strains were isolated from both tube lumen contents and biofilms.

### Multilocus sequence typing

The 7-loci MLST sequence types (ST) obtained for eight selected strains are given in Table 2. Five STs were identified across the pulsotype groups, and were internally consistent within the clonal group. Pulsotypes 1 to 4 and the unique isolate corresponded with sequence types ST2076, ST73, ST95, ST127 and ST394, respectively. Sequence types 394 and 2076 differ by one nucleotide in the *parA* allele. These strains had been isolated from two hospitals at different times; 1 October 2007, PT4 January and February 2007. All the sequence types belong to the extra-intestinal (ExPEC) pathogenic *E. coli* group B2; [38].

**Table 2:** Characterisation of *E. coli* strains isolated from neonatal nasogastric feeding tubes

Strain	1047	1050	1009	904	923	939	780	1008
	(ST2076)^a^	(ST2076)	(ST73)	(ST95)	(ST95)	(ST95)	(ST127)	(ST394)
Reaction								
Haemolysis reaction on sheep blood agar	α	α	α	α	α	α	α	β
Motility	NM	NM	NM	NM	NM	NM	NM	Motile
MLST loci *adk*	21	21	36	37	37	37	21	13
*fumC*	35	35	24	38	38	38	35	14
*gyrB*	61	61	9	19	19	19	61	19
*icd*	52	52	13	37	37	37	52	36
*mdh*	5	5	17	17	17	17	5	23
*purA*	77	77	11	11	11	11	5	11
*recA*	4	4	25	26	26	26	4	10
O-antigen^b^	O44	-	O25:K5	O1:K1	-	-	O77	O-rough
Sequence type	2076	2076	73	95	95	95	394	127

^a^Sequence type given in parenthesis, ^b^Laboratory determination by Statens Serum Institut of pulsotype representatives

### Antibiograms

Antimicrobial susceptibility of the *E. coli* strains is given in Table 3. The two *E. coli* ST2076 strains 1047 and 1050, which belong to PT1, showed resistance to the penicillin antibiotics and were susceptible to all other antibiotics. The PT2 strain 1009, belonging to ST73, was susceptible to all antibiotics. The ST95 (PT3) *E. coli* strains 904, 923 and 939 were susceptible to all antibiotics, except ampicillin. *E. coli* strain 780 (ST394, PT4) was resistance to ampicillin, and augmentin. *E. coli* strain 1008 (ST127, U) was susceptible to all antibiotics. The two closely related STs 394 and 2076 differed in their susceptibility to piperacillin and meropenem; Table 3. The *bla*_tem_ gene, conferring resistance to ampicillin, was found in *E. coli* ST95, ST394 and ST2076 isolates and none harboured *bla*_SHV_*, bla*_CTX*-M*_ or *bla*_OXA_. *E. coli* strains 1008 (ST127, U) and 1009 (ST73, PT2) did not encode any of these genes.

**Table 3:** Antibiograms of selected nasogastric tube *E. coli* isolates based on pulsotype

Antibiotic group	Antibiotic	ST2076		ST73PT2	ST95			ST394	ST127
		(PT1)^a^		(PT2)	(PT3)			(PT4)	(U)
		1047	1050	1009	904	923	939	780	1008
Cephalosporins	Cefpodoxime	S	S	S	S	S	S	S	S
	Cefotaxime	S	S	S	S	S	S	S	S
	Ceftazidime	S	S	S	S	S	S	S	S
Penicillins	Ampicillin	R	R	S	R	R	R	R	S
	Augmentin	R	R	S	S	S	S	R	S
	Piperacillin/Tazobactam	R	R	S	S	S	S	S	S
Fluoroquinolones	Ciprofloxacin	S	S	S	S	S	S	S	S
Aminoglycosides	Gentamicin	S	S	S	S	S	S	S	S
Carbapenems	Imipenem	S	S	S	S	S	S	S	S
	Meropenem	S	S	S	S	S	S	R	S
Miscellaneous	Chloramphenicol	S	S	S	S	S	S	S	S

^a^Pulsotype is given in parenthesis

### Physiological traits

Eight strains were selected as representatives of the initial thirty *E. coli* strains for further detailed study; 1047 (ST2076), 1050 (ST2076), 1009 (ST73), 904 (ST95), 923 (ST95), 939 (ST95), 780 (ST127), and 1008 (ST394). The *E. coli* strains were non-motile and showed α-haemolysis on sheep blood agar, except for strain 1008 ST394 which was motile and β-haemolytic; Table 2. The serotype of pulsotype representatives were determined by laboratory analysis (Statens Serum Institute). This revealed a range of O-types including O1:K1 (ST95) and O25:K5 (ST73); Table 2.

### Genome analysis of *E. coli* ST95 isolates

**Fig. 2:**
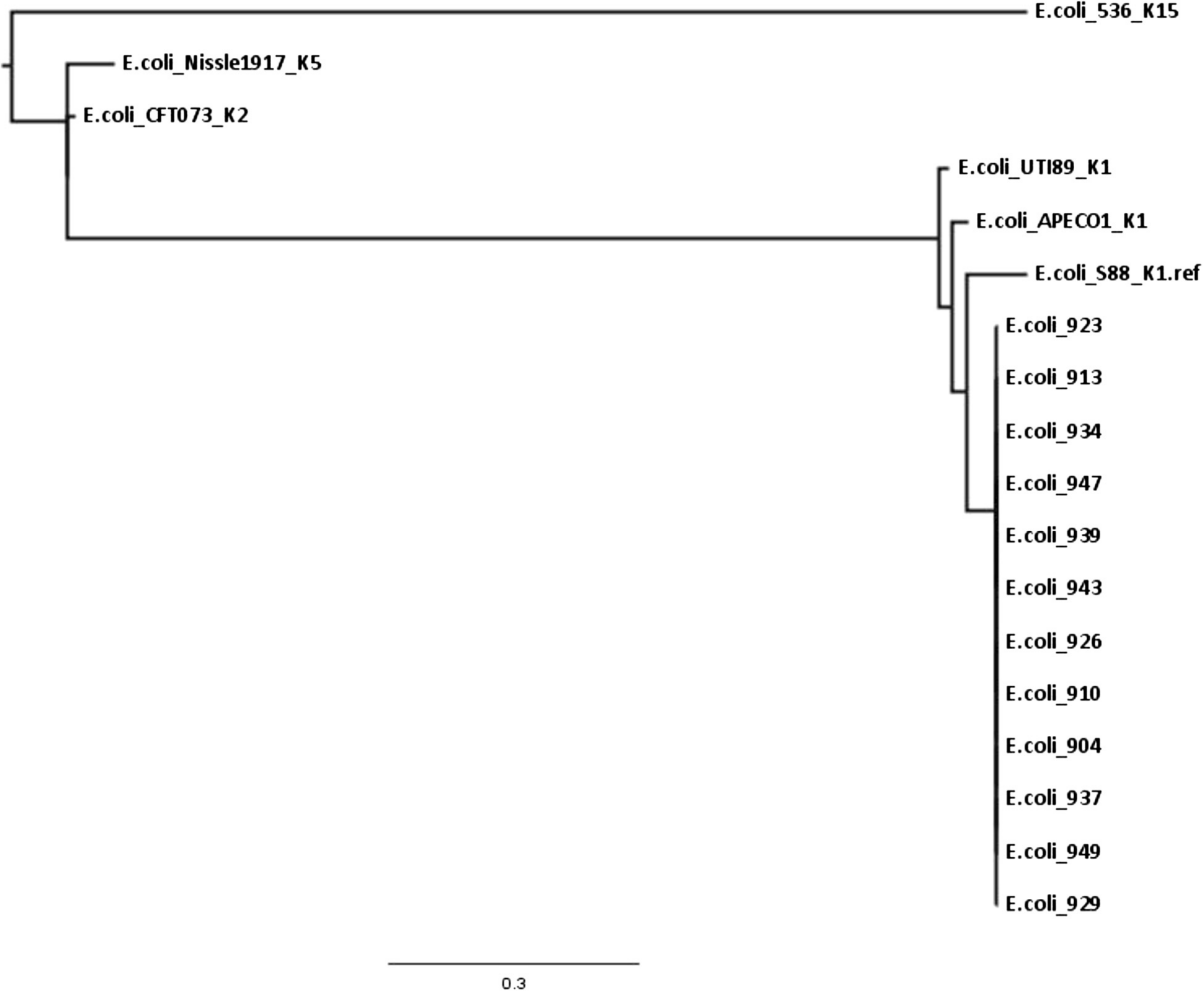
Comparative genomic alignment of *E. coli* K1 isolates with reference *E. coli* genomes

The *E. coli* K1 phylogenetic group B2 ST95 strains with indistinguishable pulsotypes (PT3) had been isolated from the feeding tubes of 11 neonates in an intensive care unit over a two week period (Fig. 1). As given above, the representative PT3 strain (904) was laboratory determined to be O1:K1; Table 2. Given the significant association of *E. coli* K1 with neonatal meningitis, all the PT3 strains were genome sequenced. Their genome size was in the order of 4997507 bp, average G + C content was 50.7 % (ranging from 49.2 to 51.3 %). The genome annotation indicated that all the *E. coli* ST95 strains were serotype O1 and capsular type K1. Whole genome alignment was performed for conformational purposes with a variety of publically available genomes of *E. coli* strains expressing different K antigens; Fig. 2. The genomes also revealed the presence of genes encoding for curli fimbriae and colanic acid which are associated with extracellular matrix production and biofilm formation.

The range of antibiotic resistance encoding genes or ORFs was predicted using the Comprehensive Antibiotic Database (CARD; http://arpcard.mcmaster.ca) and has been summarised in Table 4. This analysis revealed the *E. coli* ST95 strains had two streptomycin resistance associated genes (*strA* and *strB*) in the aminoglycoside resistance class. The number of predicted antibiotic efflux genes in the 12 *E. coli* ST95 strains varied slightly. The majority (10/12) of strains had 52 genes, whereas one had 51 and the remainder had 53. This was the only trait which distinguished between the ST95 strains. Further studies will provide a more in-depth analysis of this small variation. Also the *E. coli* ST95, ST2076, and ST127 had an additional β–lactamase gene compared with ST73 and ST394. Further detailed analysis is given in the Additional file 1: Figure S1.

**Table 4:** Number of antibiotic resistance genes or open reading frames according to antibiotic classes as predicted using Comprehensive Antibiotic resistance Database (CARD; http://arpcard.mcmaster.ca)

*E. coli* isolate	Sequence type	Aminoglycoside	β-lactamase	Sulfonamide	Polymyxin	Peptide/Bacitracin	Lincosamide	Isoniazid/Miscellaneous	Mac/lin/phe/str/lin^a^	Streptothricin	Antibiotic efflux^b^
1047	2076	1	3	1	7	1	1	1	1	0	51
1009	73	0	2	0	8	1	1	1	1	0	51
904	95	2	3	1	7	1	1	1	1	0	52
910	95	2	3	1	7	1	1	1	1	0	52
913	95	2	3	1	7	1	1	1	1	0	52
923	95	2	3	1	7	1	1	1	1	0	52
926	95	2	3	1	7	1	1	1	1	0	52
929	95	2	3	1	7	1	1	1	1	0	51
934	95	2	3	1	7	1	1	1	1	0	52
937	95	2	3	1	7	1	1	1	1	0	52
939	95	2	3	1	7	1	1	1	1	0	53
943	95	2	3	1	7	1	1	1	1	0	52
947	95	2	3	1	7	1	1	1	1	0	52
949	95	2	3	1	7	1	1	1	1	0	52
780	127	1	3	0	7	1	2	1	1	1	52
1008	394	0	2	0	7	1	2	1	1	0	52

^a^Macrolide, linezolid, phenicol, streptogramin, lincosamide

^b^Predicted genes linked to antibiotic transport system or modulation of efflux systems

### Virulence traits

The presence of 30 virulence related traits were screened using PCR. The 30 virulence genes investigated, included a range of traits including genes encoding for adhesins, invasins, capsule, toxins, siderophores and others. The presence of these genes differed depending on the strain sequence type; Table 5. For example, *E. coli* K1 ST95 strains encoded adhesin genes *fimH*, *papACEFG1,* siderophores *fyuA*, *traT* and UPEC PAI. In contrast, the ST127 *E. coli* K5 strain 1008 encoded *sfaS*, haemolysin *hlyA* and *cnf.* The UPEC PAI marker, *malX* from archetypal ExPEC strain CFT073 (serotype O6:K2:H1), was only present in STs 73, 95, and 127. The aerobactin receptor gene (*iutA*) was only in ST73 (PT2). The two closely related STs 394 and 2076 differed in the possession of *fimH* and *fvtA*; Table 5.

**Table 5:** Distribution virulence factors across selected *E. coli* isolates from nasogastric tubes based on pulsotype

			Adhesins																Invasion	Capsule					Toxins		Siderophores			Others		
*E. coli* strain	PT	ST	afa/draBC	bmaE	focG	fimH	gafD	papEF	papA	papC	nfaE	sfa/focDE	papG allele II	papG I	papG II,III	papG allele	sfaS	papG allele I	ibeA	kpsMT III	kpsMT II	k1	k5	hlyA	cnf+	cdtB	fyuA	iutA	malX	rfc	cvaC	traT
1047	PT1	2076	-	-	-	+	-	-	-	-	-	-	-	-	-	-	-	-	-	+	+	-	+	-	-	-	-	-	-	-	-	+
1050	PT1	2076	-	-	-	+	-	-	-	-	-	-	-	-	-	-	-	-	-	+	+	-	+	-	-	-	-	-	-	-	-	+
1009	PT2	73	-	-	+	+	-	+	+	+	-	+	-	+	-	+	-	-	-	-	+	-	+	+	+	-	+	+	+	-	-	-
904	PT3	95	-	-	-	+	-	+	+	+	-	-	+	+	-	-	-	-	-	-	+	+	-	-	-	-	+	-	+	-	-	+
923	PT3	95	-	-	-	+	-	+	+	+	-	-	+	+	-	-	-	-	-	-	+	+	-	-	-	-	+	-	+	-	-	+
939	PT3	95	-	-	-	+	-	+	+	+	-	-	+	+	-	-	-	-	-	-	+	+	-	-	-	-	+	-	+	-	-	+
780	PT4	394	-	-	-	-	-	-	-	-	-	-	-	-	-	-	-	-	-	+	+	-	+	-	-	-	+	-	-	-	-	+
1008	U	127	-	-	-	+	-	+	+	+	-	+	-	+	-	+	+	-	-	+	+	-	+	+	+	-	+	-	+	-	-	+

*PT* pulsotype, *U* unique, *ST* sequence type

### Adhesion, invasion and persistence in mammalian cell types

**Fig. 3:**
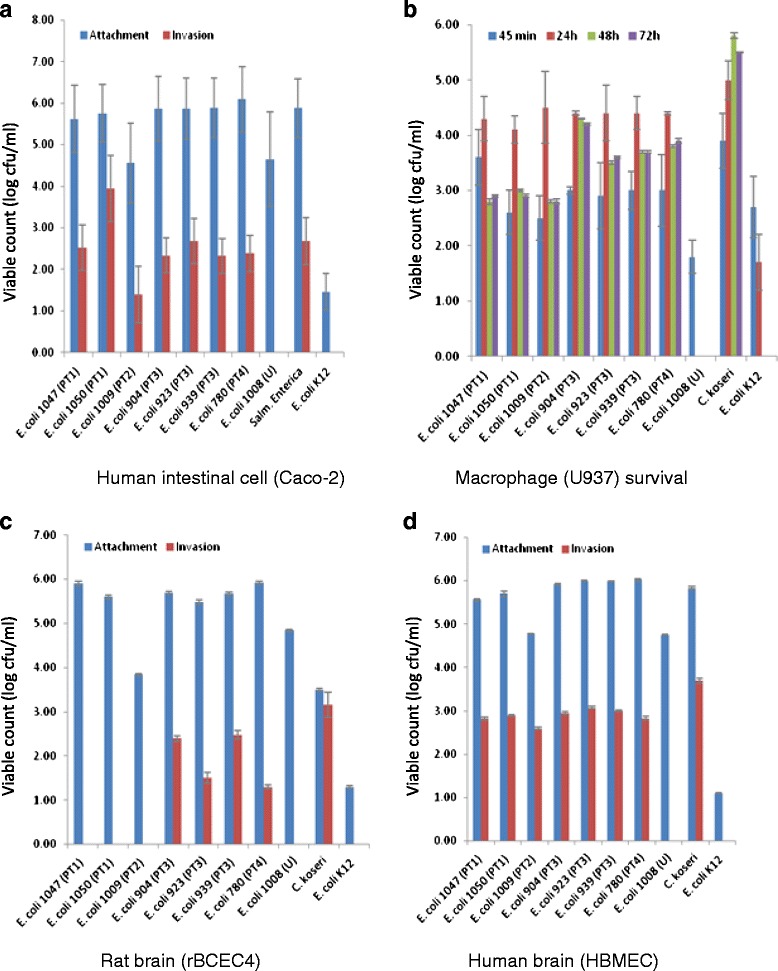
Attachment, invasion, and persistence of *E. coli* isolates in **a** intestinal **b** macrophage, **c** rat brain cells **d** human brain cells

*In vitro* tissue culture assays showed that *E. coli* sequence types varied in their ability to attach and invade mammalian cell lines; Fig. 3. *E. coli* K1 ST95 attached and invaded intestinal cells (Caco-2), and both human and rat brain cell lines; HBMCE and rBCEC4. They also persisted for 48 h in U937 macrophages; Fig. 3. *E. coli* STs 73, 394 and 2076 also persisted in macrophages and invaded Caco-2 and human brain cells, but only ST394 invaded rat brain cells. *E. coli* ST127 was notable as it did not invade any cell lines. Nearly all strains of *E. coli* showed an aggregative attachment pattern on Caco-2 and Hep-2 cell lines. The exception was *E. coli* ST127 strain 1008 which showed a diffuse attachment pattern on both cell lines. This strain is also O-rough antigen type; Table 2. One-way ANOVA statistical analysis demonstrated that all strains attached significantly more than *E. coli* K12 (p ≤ 0.001).

## Discussion and conclusions

Microbial colonisation of the neonate starts at birth, or even sooner through the meconium [39]. This initial flora is largely commensal but may include *E. coli* pathovars [40]. Hurrell et al. [19] has already given a general overview of the *Enterobacteriacae* isolates from bacterial biofilms inside neonatal nasogastric feeding tubes collected from these two hospitals. Those studies included electron micrographs of multi-organism (bacteria and fungi) biofilms inside the used feeding tubes. *E. coli* was isolated from 29 % (*n* = 129) of these tubes. However, in that study, the *E. coli* strains had not been genotyped to determine if there was a common source.

Our follow-up study shows, as expected, that the same pulsotype strains were isolated from both the residual liquid of the lumen and inner surface biofilm from 66 % of tubes; Table 1. For example, PT1 were three strains from one neonate, which had been isolated on the same day. This neonate had been fed ready-to-feed formula. Given that this is a sterile product, the source of the *E. coli* strains inside the feeding tube is uncertain. This issue has already been considered by Hurrell et al. [19] who proposed that a possible secondary source of the enteral tube flora was the throat due to gastroesophageal reflux. In preterm neonates this occurs 3–5 times per hour when the lower oesophageal sphincter relaxes. This would increase the exposure of the feeding tube to the throat flora.

However, Fig. 1 also shows that the 30 *E. coli* isolates only formed five pulsotypes, and therefore multiple indistinguishable strains had been isolated from different neonates over a one year period. PT4 strains had been isolated from three neonates over a 4 week period. These neonates had all received breast milk, and two had also received reconstituted infant formula. This demonstrates the possible dissemination of strains in the neonatal intensive care unit. Of particular significance was pulsotype 3 which was composed of 19 indistinguishable *E. coli* strains from 11 neonates on different feeding regimes, over a two week period; Table 1. This reinforces the probability that strains were acquired due to dispersion within the intensive care unit by carers and the environment and not a specific feed source such as contaminated infant formula.

MLST revealed that each pulsotype corresponded with a unique sequence type. It is noted that although ST394 and ST2076 only differ in one nucleotide in the *parA* allele, that the strains differed in their antibiotic susceptibilities and virulence; Table 2. The *E. coli* ST127 strain 1008 was the only strain which was motile, and also showed β-haemolysis on sheep blood agar; Table 2.

Since the clinical representation of the neonates in the study was not available, the potential pathogenicity of the strains was assessed using both genetic analysis for virulence traits (PCR-probes, and genome sequence analysis) as well as *in vitro* tissue culture. *E. coli* K1 translocates from the neonatal intestines to the bloodstream, where they multiply and cross the blood–brain barrier by invading the brain microvascular endothelial cells. These steps were investigated using attachment and invasion studies of human colonic carcinoma epithelial cells (Caco-2), rat blood brain barrier cells (rBECE4) and human brain microvascular endothelial cells (HBMEC) tissue culture cells; Fig. 3. Macrophage survival was studied using the (U937) cell line of human monocyte cells. These assays revealed there was considerable variation in the presence of virulence traits and *in vitro* pathogenicity according to the *E. coli* sequence type.

The three *E. coli* K1 ST95 strains were notable for their ability to attach and invade intestinal and both human and rat brain cells at levels comparable to *Salmonella enterica* and *C. koseri,* respectively; Fig. 3a, c, d. Macrophage uptake and persistence was comparable to *C. koseri*; Fig. 3b. The three other sequence types (ST394, ST73, ST2076) also attached and invaded human intestinal and brain cells, and ST394 was also able to invade rat brain cells.

These five sequence types are in the ExPEC biogroup B2, and combining the results of the motility assay with serotyping showed that the ST95 strains were *E. coli* O1:K1:NM. This group is of high significance due to their strong association with neonatal meningitis, and on this occasion 19 indistinguishable strains had been isolated from the tubes of 11 neonates. *E. coli* phylogroup B2 ExPEC strains of serotypes O1, O2, O18, and O45 are most frequently in ST95, and have been a focus of considerable research in recent years [12–14, 41,42]. In order to assess the virulence potential of the strains, a total of 30 virulence genes were screened for. These included adhesins, invasins, capsule, toxins, siderophores and others commonly associated with neonatal meningitic *E. coli* (NMEC), avian pathogenic *E. coli* (APEC) and uropathogenic *E. coli* (UPEC) [28].

The presence of the virulence related genes differed depending on the sequence type; Table 5. For example, *E. coli* O1:K1:NM ST95 strains encoded adhesin genes *fimH*, *papACEFGI, papG allele II,* siderophores *fyuA* (yersiniabactin receptor), and UPEC pathogenicity associated island (PAI) marker (*malX*) as well as the serum resistance associated gene *traT*. However despite the attachment and invasion of human and rat brain cells (Fig. 3), the ST95 strain however did not encode for *ibeA* or *sfaS*. Similarly Johnson et al. [17] reported their occurrence in only 33 % and 59 % of NMEC strains (*n* = 70), respectively. In contrast, the β–haemolytic ST127 *E. coli* K5 strain 1008 which did not attach or invade any cell line encoded for the *sfaS* adhesin as well as the haemolysin encoded by *hlyA* and the cytotoxic necrotizing factor (*cnf). MalX,* a marker for a UPEC PAI from the archetypal ExPEC strain CFT073 (serotype O6:K2:H1) [43] was present in sequence types 73, 95, and 127. A fuller description of the *E. coli* genomes derived from this study will be given in a separate publication.

Mora et al. [38] reviewed the source and virulence profiles of 59 ExPEC O1:K1:H7/NM ST95 strains of animal and human origin, recovered from different dates and geographic sources. They reported that some APEC isolates may act as potential pathogens for humans from poultry, suggesting no host specificity for this type of isolate. In contrast, the strains in this study had been isolated from preterm neonates in isolation units who had been fed breast milk and infant formula. Microbiological analysis of the feeds and microbial carriage by staff was not assessed at the time by Hurrell et al. [19]. Therefore the source of these *E. coli* K1 strains is currently uncertain.

*E. coli* K1 are the second most common cause of severe neonatal infections after Group B streptococcal (GBS) meningitis [44]. Although 85 % of infected neonates recover, this is often not as full as would occur with older infants and children. Sources and dissemination of such pathogenic organisms needs further investigation, especially since *E. coli* K1 causes 80 % of neonatal meningitis cases. Neonates acquire their initial flora at birth from the mother, environment and other carers. Maternal to child transmission of *E. coli* has been reported, and has been linked to late-onset neonatal infection [45–47]. There is also the possible transmission of *E. coli* K1 by nurses’ hands [40]. In addition, recent microbiome studies have indicated the possible dispersion of bacteria in the neonatal intensive care units [48]. It should also be noted that *E. coli* ST73, ST394 and ST2076 strains demonstrated the ability to invade human cells lines and therefore their occurrence in nasogastric feeding tubes may additionally pose a pathogenic risk towards neonates.

Due to confidentiality reasons, the clinical condition of the specific neonates from whom the *E. coli* strains in this study were isolated is not available. However during this collection period there were four cases of *E. coli* infection. It should be noted that the genomic analysis showed the *E. coli* K1 strains (ST95) had two streptomycin resistance genes belonging to the aminoglycoside class antibiotics (Table 4). This could be of clinical significance since aminoglycoside antibiotics such as gentamicin are regularly used as 1^st^ and 2^nd^ line combinations on NICUs. Since the patients’ details and isolates were not available for analysis, no direct causal infection route from the nasogastric tube can be made. Attribution of the source of the *E. coli* K1 ST95 is not feasible as there no environmental sampling or screening of carriage by staff or mothers. Nevertheless given the indistinguishable strains were obtained from neonates on different feeding regimes it seems probable that strains were disseminated in the NICU by carers and the environment, and not directly from a single feeding source.

### Ethics statement

Isolates from this study were obtained by culturing stock isolates. All clinical data are taken from a previous publication [19].

## Additional file

Additional file 1: Figure S1. Genomic analysis of *E. coli* sequence types using the Comprehensive Antibiotic Database (CARD: http://arpcard.mcmaster.ca). (DOC 1740 kb)

## Abbreviations

Caco-2: Human colonic carcinoma epithelial cells
CFU/ml: Colony forming units per millilitre
ExPEC: Extra-intestinal pathogenic *E. coli*
GBS: Group B streptococci
HBMEC: Human brain microvascular endothelial cells
MLST: Multilocus sequence typing
NICU: Neonatal intensive care unit
NM: Non-motile
NMEC: Neonatal meningitic *E. coli*
PAI: Pathogenicity associated island
PCR: Polymerase chain reactions
PFGE: Pulsed-field gel electrophoresis
PT: Pulsotype
rBECE4: Rat blood brain barrier cells
ST: Sequence type
UPEC: Urinary pathogenic *E. coli*
UPGMA: Unweighted pair group method with arithmetic mean
