# Supplementary material for: The molecular characterisation of Escherichia coli K1 isolated from neonatal nasogastric feeding tubes
Source: BMC Infect Dis. 2015 Oct 26;15:449. doi: 10.1186/s12879-015-1210-7 (PMC4620641; doi:10.1186/s12879-015-1210-7)

Additional file 1: Figure S1. Genomic analysis of *E. coli* sequence types using the Comprehensive Antibiotic Database (CARD: http://arpcard.mcmaster.ca).


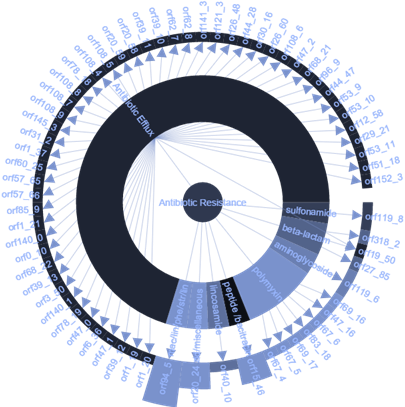

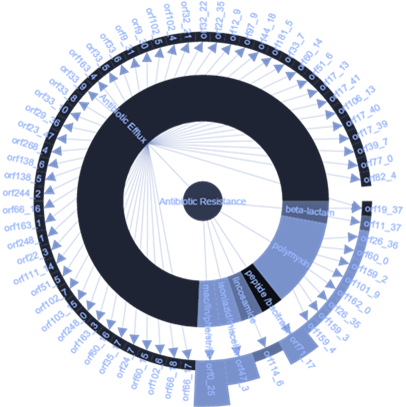
(a) *E. coli* 1047 (ST2076) (b) *E. coli* 1009 (ST73)


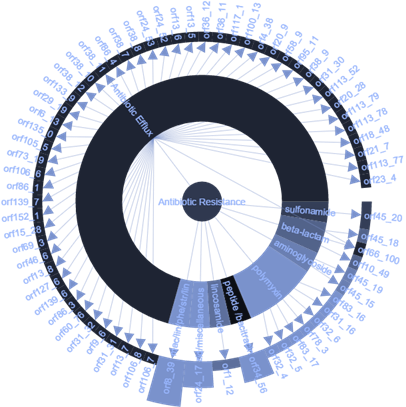

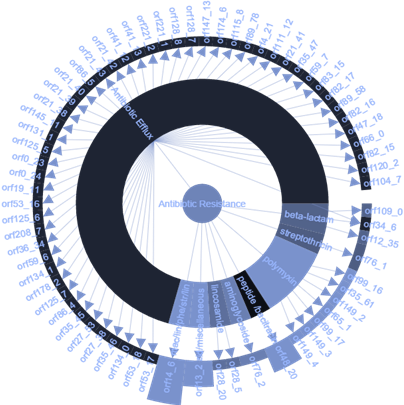
(c) *E. coli* 904 (ST95) (d) *E. coli* 780 (ST127)

(e) *E. coli* 1008


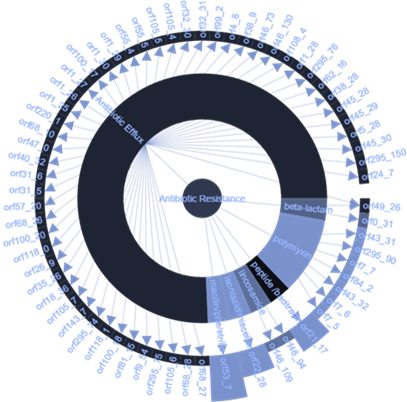

Supplement: Additional file 1: Figure S1. — Genomic analysis of E. coli sequence types using the Comprehensive Antibiotic Database (CARD: http://arpcard.mcmaster.ca). (DOC 1740 kb) [file 12879_2015_1210_MOESM1_ESM.doc]
